# Antenatal Depression Risk and Mental Health Literacy: A Correlational Study Among Second- and Third-Trimester Pregnancies

**DOI:** 10.3390/healthcare14162545

**Published:** 2026-08-14

**Authors:** Ege Miray Topcu, Emel Öztürk Turgut, Gizem Beycan Ekitli

**Affiliations:** 1 Nursing Department, Health Sciences Faculty, Karadeniz Technical University, 61080 Trabzon, Türkiye; egemiraytopcu@ktu.edu.tr; 2 Psychiatric Nursing Department, Ege University Nursing Faculty, 35100 İzmir, Türkiye; emel.ozturk@ege.edu.tr

**Keywords:** antenatal depression, mental health literacy, pregnancy, antenatal care, maternal mental health

## Abstract

**Highlights:**

**What are the main findings?**

- Overall mental health literacy was not associated with depressive symptom severity or EPDS ≥ 10 screening positivity; however, third-trimester and unplanned pregnancies were associated with higher odds of screening positivity.
- Knowledge- and resource-oriented literacy showed opposing, model-dependent associations with symptom severity, with the resource-oriented association attenuated when examined separately.

**What are the implications of the main findings?**

- Antenatal care should prioritise routine symptom screening rather than assuming lower risk among women with greater mental health literacy.
- Subscale-specific findings require replication before informing mental health literacy interventions.

**Abstract:**

**Background/Objectives:** Antenatal depressive symptoms are common but frequently under-recognized. Although mental health literacy may support symptom recognition and help-seeking, its relationship with antenatal depressive symptoms remains unclear. This study examined the association between overall mental health literacy and antenatal depressive symptoms among women in the second and third trimesters of pregnancy. **Methods:** This cross-sectional correlational study included 568 pregnant women consecutively recruited from a university hospital’s obstetrics and gynecology outpatient clinics. The Edinburgh Postnatal Depression Scale (EPDS) and Mental Health Literacy Scale (MHLS) were administered. Spearman correlation and multivariable linear and logistic regression were used; multiple imputation handled missing covariates. **Results:** The mean EPDS score was 8.92 ± 5.68, and 41.9% (*n* = 238) screened positive for elevated depressive symptoms at the EPDS ≥ 10 threshold. A one-standard-deviation increase in the MHLS total score was associated with neither EPDS total scores (B = 0.011, 95% CI: −0.451 to 0.473, *p* = 0.963) nor EPDS ≥ 10 screening positivity (aOR = 1.093, 95% CI: 0.911 to 1.312, *p* = 0.337). Unplanned pregnancy (aOR = 1.975, *p* = 0.001) and third-trimester status (aOR = 1.774, *p* = 0.014) were associated with higher odds of screening positivity. In exploratory joint analyses, knowledge-oriented literacy was positively and resource-oriented literacy negatively associated with EPDS scores; both remained significant after Holm correction. The resource-oriented association was no longer significant when examined separately. **Conclusions:** Overall mental health literacy was not associated with antenatal depressive symptom severity or screening positivity. Opposing, model-dependent subscale findings require replication. These findings support assessing depressive symptoms independently of mental health literacy scores and considering pregnancy planning and gestational stage during antenatal psychosocial assessment.

## 1. Introduction

Health literacy refers to individuals’ knowledge and skills in accessing health services and reliable resources to protect and improve their health and well-being. It empowers individuals to participate actively in managing their own health and the health of those close to them [1]. Similarly, mental health literacy encompasses the knowledge and skills required to enhance mental well-being, recognize symptoms of mental disorders, understand attitudes toward mental illness and identify mental health support resources. It is a crucial tool for promoting public mental health and addressing mental illnesses [2,3,4]. During pregnancy, when physical and psychological needs are closely intertwined, mental health literacy may serve as an important individual resource by helping women recognize changes in their emotional well-being, access reliable information and support resources, and seek professional help when needed [5,6,7].

Pregnancy is a major life transition marked by enduring changes and transition to motherhood. Research and public health reports indicate that women may be at risk for mental health problems during both pregnancy and the postpartum period [8,9]. Ou et al. (2025) estimated that approximately one in ten women experienced co-occurring anxiety and depression during the perinatal period [9]. Meta-analyses have estimated the prevalence of depression during pregnancy to range from 19.5% to 34.0% [10,11,12,13]. In a recent study conducted in Türkiye, 34.7% of primigravid women screened positive for postpartum depression risk on the Edinburgh Postnatal Depression Scale (EPDS), whereas 9.4% exhibited moderate-to-severe depressive symptoms on the Beck Depression Inventory (BDI) [14]. Antenatal depression has been associated with adverse birth outcomes [15] and an approximately fourfold higher risk of postpartum depression [16]. These findings underscore the importance of early identification and appropriate psychosocial support during pregnancy [17].

Antenatal depression is associated with a range of biological, obstetric, and, in particular, psychosocial factors. A history of depression and other pre-pregnancy health problems, low income, difficulties in partner relationships, inadequate social support, unplanned pregnancy, a history of violence, stressful life events, and previous pregnancy and childbirth experiences have been identified as major factors associated with antenatal depression [10,11,12,13,18]. These findings indicate that women’s psychosocial circumstances and mental health needs should be considered together when assessing the risk of antenatal depression. Nevertheless, women experiencing mental health problems during the antenatal period have been reported to rely primarily on support from people close to them and to make limited use of professional perinatal mental health services. A major barrier to seeking help during pregnancy is a lack of knowledge about mental health problems, their symptoms, and available support resources [6,7]. Recent evidence similarly indicates that a substantial proportion of pregnant and postpartum women do not use mental health services even after screening positive, with limited awareness of available services, stigma, financial pressure, and insufficient partner support identified as important barriers to service utilization [15]. Mental health literacy among women and their close social networks is therefore important for recognizing changes in women’s mental health, considering these changes without stigma, and accessing appropriate sources of support [7,19].

Although the perinatal literature has examined several dimensions of depression and mental health literacy, the direct association between mental health literacy and antenatal depression risk has received limited attention. In a study of women during the perinatal period, Fonseca et al. (2017) found clinically significant psychopathological symptoms in 34% of participants and reported a moderate level of depression literacy [5]. However, the association between depression literacy and psychopathological symptom risk was not examined. Similarly, in a study conducted among postpartum women in India, only 50.7% of mothers demonstrated an adequate level of knowledge about postpartum depression, and postpartum depression literacy was associated with age, income, and employment status [20]. These findings indicate that depression-related literacy may vary according to sociodemographic characteristics, but they do not clarify whether general mental health literacy is associated with depressive symptoms during pregnancy. A recent cluster-randomized trial found that antenatal psychoeducation improved overall postpartum depression literacy, symptom recognition, and access to information but did not significantly improve attitudes toward seeking professional help [16]. Together, existing studies indicate gaps in perinatal mental health knowledge, service awareness, and professional help-seeking [19,20,21,22]. However, it remains unclear whether overall mental health literacy is associated with antenatal depressive symptom severity or screening positivity independently of relevant sociodemographic and obstetric factors.

Mental health literacy is not a unitary construct. It is commonly conceptualized as comprising knowledge-oriented literacy (factual understanding of mental disorders and their symptoms), belief-oriented literacy (attitudes and stigma-related beliefs) and resource-oriented literacy (awareness of where and how to obtain help) [2,4]. These dimensions may not operate uniformly: recognizing symptoms does not necessarily translate into help-seeking, and knowing where to seek support may matter more than factual knowledge alone. Consistent with this, the cluster-randomized trial cited above improved symptom recognition and access to information without changing attitudes towards professional help-seeking [16]. Given the limited prior evidence, domain-specific analyses were undertaken post hoc and interpreted as exploratory.

Together, these findings underscore the importance of assessing mental health literacy among women during the perinatal period while also highlighting the need to clarify its association with antenatal depression risk. Therefore, this study aimed to describe antenatal depressive symptom and mental health literacy levels among pregnant women and to examine whether the MHLS total score was associated with EPDS total scores. Clarifying this relationship may inform early psychosocial assessment and mental health promotion practices in antenatal care. In the present study, antenatal depression risk refers to elevated depressive symptoms identified through EPDS screening rather than a DSM-5 diagnosis of major depressive disorder with peripartum onset [17,18,19,20,21,22,23]. The prespecified primary hypothesis (H1) was that the MHLS total score would be associated with the EPDS total score. A secondary analysis examined whether the MHLS total score was associated with EPDS ≥ 10 screening positivity after adjustment for relevant sociodemographic, obstetric, and health-related factors.

## 2. Methods

### 2.1. Research Design

This study followed a cross-sectional, correlational design and was reported in accordance with the Strengthening the Reporting of Observational Studies in Epidemiology (STROBE) guideline.

### 2.2. Participants and Setting

The study was conducted in the obstetrics and gynecology outpatient clinics of a university hospital in western Türkiye. The study population comprised pregnant women who attended these clinics for antenatal care and routine pregnancy follow-up between July 2023 and June 2024.

Women were eligible if they were aged 18 years or older, were at least 20 weeks pregnant, were able to read and understand Turkish, had the physical and cognitive capacity to complete the data collection forms independently, and provided written informed consent. Women who were less than 20 weeks pregnant were not included for two reasons: the risk of pregnancy loss is higher before 20 weeks, and the national screening tests and routine ultrasound assessments are completed during this period. The study was therefore restricted to the second and third trimesters. Exclusion criteria were a decision to terminate the pregnancy, the onset of labor, and any cognitive, communicative or decision-making difficulty that would prevent the forms from being understood or completed independently.

Participants were recruited using non-probability consecutive sampling. Throughout the predefined data collection period (July 2023 to June 2024), all pregnant women who attended the clinic, in order of presentation, and met the eligibility criteria were invited to take part, and those who gave written informed consent were enrolled without further selection. Recruitment ended when the predefined data collection period closed rather than when a target sample size was reached. The numbers of women approached and declining participation were not systematically recorded.

Because effect size estimates from studies examining this specific combination of variables were limited, a medium effect size was assumed for the a priori calculation. The sample size was estimated using G*Power (version 3.1.9.7; Heinrich Heine University Düsseldorf, Düsseldorf, Germany) for a two-tailed correlation analysis, assuming r = 0.30, α = 0.05 and 80% statistical power; the minimum required sample size was 84 participants. The achieved sample of 568 substantially exceeded this minimum. The adequacy of the multivariable models was assessed separately using the events-per-variable criterion (see Section 2.5).

During the data collection period, 584 eligible and consenting women completed the data collection forms. At the data-checking stage, the forms of 12 participants were excluded because of incomplete scale responses. Participants who did not respond to all scale items were excluded from the analysis; consequently, the analytical sample contained no missing data on either scale. Missing data on sociodemographic and obstetric variables were handled as described in Section 2.5. Four participants were found to have completed the form twice; in each case the duplicate record was removed and the first record retained. The final analytical sample therefore comprised 568 participants.

### 2.3. Data Collection

Data were collected cross-sectionally from July 2023 to June 2024. The study was introduced in person to pregnant women visiting the outpatient clinic and those who met the inclusion criteria completed the survey forms independently. The data collection process took approximately 15 min per participant. No follow-up or monitoring measures were conducted in the study.

#### Measures

A pilot study with 10 participants was conducted to evaluate the clarity of the questions, the usability of the data collection tool and the overall data collection process. Feedback from this pilot study was used to finalize the data collection instruments. The data collection tools were designed for use in any setting where participants could read and understand the content while maintaining their privacy. The 10 pilot participants were not included in the recruitment totals or the final analytical sample.

Demographic Information Form: Created by the researcher, this form consists of 16 questions designed to assess participants’ sociodemographic characteristics—such as age, marital status, education and income level—and pregnancy-related factors (Table 1).

Edinburgh Postnatal Depression Scale (EPDS): Developed by Cox et al. (1987), the validity and reliability of this scale were established by Engindeniz et al. (1996) [17,24]. The scale is used both nationally and internationally to assess the risk of postpartum and antenatal depression [25,26]. It consists of 10 items, rated on a 4-point Likert scale, ranging from 0 to 3, with 7 items reverse-coded. Engindeniz et al. (1996) [24] found the reliability of the scale to be 0.80. A higher total score on the EPDS indicates a higher level of depressive symptoms [25]. In the present study, scores of ≥10 were interpreted as indicating screening positivity for elevated antenatal depressive symptoms rather than a clinical diagnosis.

Mental Health Literacy Scale (MHLS): Developed by Jung et al. (2016) and adapted into Turkish by Göktaş et al. (2019), the MHLS includes 3 sub-dimensions and 22 items (range:0–22) [2,4]. The knowledge-oriented and belief-oriented sub-dimensions consist of 18 items that are rated on a six-point Likert scale, with responses ranging from ‘strongly agree’, ‘agree’, ‘neutral’, ‘disagree’, ‘strongly disagree’ and ‘don’t know’. The resource-oriented sub-dimension uses 4 items answered with ‘yes’ or ‘no’. ‘Strongly agree’, ‘agree’ and ‘yes’ are scored as ‘1 point’, while other responses received ‘0 points’. Two items were reverse-coded. The scale has no cut-off score; higher scores in the overall scale and each sub-dimension indicate greater mental health literacy. The original study reported a reliability of 0.71 [2], while this study found a Cronbach’s alpha coefficient of 0.75.

### 2.4. Ethical Considerations

This study was approved by the Ethics Committee of Ege University for Medical Research (Approval Decision No. 23-6.1T/66, 5 July 2023). Institutional permission was also obtained from the relevant institution. The study was conducted in accordance with the principles of the Declaration of Helsinki. All participants provided written informed consent before data collection. During data collection, pregnant women identified as being at high risk of depression by the EPDS, those indicating a risk of self-harm (item 10) and those exhibiting symptoms of depression were reported to the healthcare team at the clinic, who then requested an urgent psychiatric consultation for these participants. Additionally, participants were given a link to access the ‘Pregnancy Information Class Education Book’, published by the Public Health Institution of Türkiye (2014), which contains information on pregnancy, childbirth and the postpartum period [3].

### 2.5. Data Analysis

All analyses were performed using IBM SPSS Statistics for Windows, version 26.0, and statistical significance was set at *p* < 0.05. Continuous variables were summarized as means, standard deviations, minimum and maximum values, and categorical variables as frequencies and percentages. The raw distributions of the scale scores were assessed using the Kolmogorov–Smirnov test together with histograms; because this test is overly sensitive to small departures from normality in large samples, graphical inspection was used alongside it.

Because the raw scale score distributions departed from normality, unadjusted group comparisons were performed using the Mann–Whitney U and Kruskal–Wallis tests, and categorical variables were compared using the chi-square test. Bivariate relationships between the EPDS and the total and subscale scores of the Mental Health Literacy Scale were examined using Spearman’s correlation coefficient. Coefficients were classified according to the ranges proposed by Evans (1996), with values between 0.00 and 0.19 regarded as very weak [27].

The study was not grounded in a diagnostic classification framework; the hypotheses were formulated to examine associations along a continuum, consistent with the dimensional nature of constructs such as antenatal depressive symptoms [28,29]. EPDS scores were therefore analyzed in two complementary ways. In the primary analysis, the EPDS total score was treated as a continuous outcome. In a second set of models, EPDS scores were dichotomized at the ≥10 screening threshold in order to identify variables independently associated with screening positivity. The ≥10 threshold was selected a priori as a high-sensitivity screening cut-off rather than as a diagnostic criterion. In an individual participant data meta-analysis including both pregnant and postpartum women, sensitivity and specificity against semi-structured diagnostic interviews were 0.85 and 0.84 at the ≥10 threshold and 0.66 and 0.95 at the ≥13 threshold, and accuracy was reported to be similar in pregnant and postpartum subgroups [23]. Because the aim of the secondary screening analysis was to identify elevated symptom risk rather than to establish a diagnosis, the more sensitive threshold was used. The ≥13 threshold was examined in sensitivity analyses as a more specific screening threshold. At neither threshold does the EPDS establish a DSM-5 diagnosis; scores at or above a threshold were interpreted as indicating elevated antenatal depressive symptom risk rather than clinical depression. The association between the MHLS total score and the EPDS total score represented the prespecified primary hypothesis. The EPDS ≥ 10 model was treated as a secondary screening analysis. Multivariable adjustment and sensitivity analyses were added post hoc during manuscript revision and were interpreted accordingly.

Missing data were confined to sociodemographic and obstetric covariates, with no missing values on either scale or on any outcome; 47 participants (8.3%) had missing data on at least one model variable. Multiple imputation was conducted under the missing-at-random assumption. Multiple imputation by fully conditional specification generated 20 datasets with a maximum of 10 iterations, using predictive mean matching for continuous variables and logistic models for categorical variables. All model variables were included in the imputation model, and estimates were pooled using Rubin’s rules.

All models are specified in Supplementary Table S1. Mental health literacy total and subscale scores were standardized using the means and standard deviations observed in the original dataset and were interpreted per one standard deviation increase. All models were adjusted for age, number of pregnancies, previous miscarriage or pregnancy loss, education level, income level, trimester, pregnancy planning status, current psychiatric disorder and current physical disorder. Covariates were selected a priori on the basis of the literature and clinical relevance and entered simultaneously; selection was not based on univariate significance, and stepwise selection was not used. The literate and primary school education categories were combined to maintain adequate cell sizes. Linear regression results are reported as unstandardized coefficients (B) with standard errors, 95% confidence intervals and p values, and logistic regression results as adjusted odds ratios (aOR) with 95% confidence intervals. With 238 participants screening positive and 13 parameters, approximately 18 events per parameter were obtained. For the EPDS ≥ 13 sensitivity model, 141 screening-positive events provided approximately 11 events per parameter. Model fit was assessed using the Hosmer–Lemeshow test and Nagelkerke R^2^, reported from the complete-case analysis because fit statistics cannot be pooled under Rubin’s rules. Convergence was checked for all logistic regression models.

Because normality of the raw outcome is not an assumption of linear regression, model adequacy was assessed through the residuals: a histogram and normal P–P plot of standardized residuals, a scatterplot of standardized residuals against predicted values, tolerance and variance inflation factors, and standardized residuals, leverage values and Cook’s distance for influential observations. A significant Kolmogorov–Smirnov test was not in itself taken as a criterion for not using linear regression. Robustness was examined through the sensitivity analyses listed in Table S1.

Subscale analyses were post hoc and exploratory. The three subscale scores were standardized and entered simultaneously without the total score, and the three coefficients within each joint model were adjusted using the Holm method. To assess whether these associations depended on mutual adjustment among the subscales, each standardized subscale was subsequently examined in a separate sensitivity model using the same covariates. The primary hypothesis concerned the association between the mental health literacy total score and the EPDS total score; apart from the subscale analyses, no correction for multiple comparisons was applied.

## 3. Results

The mean age of the participants was 29.03 ± 5.49 years (range: 18–44), and 97.5% (*n* = 554) were married. Among participants with available data, 37.2% (*n* = 210) were high school graduates and 48.9% (*n* = 273) reported that their income was equal to their expenses. Regarding pregnancy characteristics, 33.9% (*n* = 187) were experiencing their first pregnancy, 78.7% (*n* = 439) were in the third trimester, and 67.8% (*n* = 381) reported no previous miscarriage or pregnancy loss (Table 1).

### 3.1. Antenatal Depressive Symptoms

The mean EPDS score was 8.92 ± 5.68 (range 0–30), and 41.9% (*n* = 238) scored at or above the ≥10 screening threshold, indicating elevated antenatal depressive symptom risk. In unadjusted comparisons, significantly higher EPDS scores were observed among participants whose income was lower than their expenses, those in the third trimester and those with unplanned pregnancies (Table 2).

### 3.2. Mental Health Literacy Scale Scores

The mean MHLS total score was 12.90 ± 4.30 (range 0–22). In unadjusted comparisons, the total score differed only by education level, with higher education associated with higher mental health literacy (KW = 30.470, *p* = 0.001). At the subscale level, knowledge-oriented and resource-oriented scores also differed by education level (KW = 64.115, *p* = 0.001 and KW = 11.620, *p* = 0.003, respectively). Knowledge-oriented scores were higher and belief-oriented scores lower among participants currently using medication (*p* = 0.033 and *p* = 0.013, respectively), and resource-oriented scores were highest among women experiencing their first pregnancy (KW = 7.834, *p* = 0.020). No other characteristic was associated with total or subscale scores (Table 3).

### 3.3. Bivariate Associations Between Mental Health Literacy and Depressive Symptoms

Neither the MHLS total score (rho = −0.014, *p* = 0.438) nor any of its subscales was significantly correlated with EPDS total scores (knowledge-oriented rho = 0.040, *p* = 0.345; belief-oriented rho = −0.023, *p* = 0.585; resource-oriented rho = −0.063, *p* = 0.132). All coefficients fell within the range classified as very weak (Table 4).

### 3.4. Factors Independently Associated with Depressive Symptoms and Screening Positivity

In the multivariable linear regression, a one standard deviation increase in the MHLS total score was not associated with EPDS total scores after adjustment (B = 0.011, SE = 0.236, 95% CI: −0.451 to 0.473, *p* = 0.963). Being in the third trimester (B = 1.572, 95% CI: 0.452 to 2.693, *p* = 0.006), having an unplanned pregnancy (B = 2.127, 95% CI: 1.100 to 3.154, *p* < 0.001) and having an income lower than expenses (B = 1.548, 95% CI: 0.202 to 2.894, *p* = 0.024) were associated with higher EPDS scores. Participants whose income equaled their expenses had lower EPDS scores than those whose income exceeded their expenses, although this association was of borderline significance (B = −1.280, 95% CI: −2.555 to −0.006, *p* = 0.049). No other covariate was significantly associated with EPDS scores (Table 5). The prespecified primary hypothesis (H1) was therefore not supported.

In the multivariable logistic regression, a one standard deviation increase in the MHLS total score was not associated with EPDS ≥ 10 screening positivity (aOR = 1.093, 95% CI: 0.911 to 1.312, *p* = 0.337). The odds of screening positivity were higher among participants in the third trimester (aOR = 1.774, 95% CI: 1.123 to 2.805, *p* = 0.014) and among those with unplanned pregnancies (aOR = 1.975, 95% CI: 1.330 to 2.935, *p* = 0.001). No other variable was independently associated with screening positivity. In the complete-case analysis, fit statistics indicated acceptable calibration (Hosmer–Lemeshow χ^2^(8) = 5.926, *p* = 0.656; Nagelkerke R^2^ = 0.135) (Table 6). The secondary screening analysis likewise provided no evidence of an association between the MHLS total score and EPDS ≥10 screening positivity.

### 3.5. Sensitivity Analyses

In the sensitivity analysis using the more specific EPDS ≥ 13 threshold, mental health literacy was again not associated with screening positivity (aOR = 0.906, 95% CI: 0.737 to 1.114, *p* = 0.350). The association with unplanned pregnancy was maintained (aOR = 1.964, 95% CI: 1.271 to 3.035, *p* = 0.002), whereas the association with the third trimester did not reach statistical significance at this more specific threshold, which included fewer screening-positive events (aOR = 1.603, 95% CI: 0.933 to 2.754, *p* = 0.088). Complete-case fit statistics for this model also indicated acceptable calibration (Hosmer–Lemeshow χ^2^(8) = 10.808, *p* = 0.213; Nagelkerke R^2^ = 0.124).

In the complete-case analysis (*n* = 521), mental health literacy remained unassociated with EPDS total scores (B = 0.134, 95% CI: −0.339 to 0.608, *p* = 0.578), while the associations with the third trimester (B = 1.463, 95% CI: 0.318 to 2.609, *p* = 0.012), unplanned pregnancy (B = 2.261, 95% CI: 1.215 to 3.307, *p* < 0.001) and income lower than expenses (B = 2.137, 95% CI: 0.749 to 3.524, *p* = 0.003) were retained; the model was significant overall [F(13, 507) = 5.667, *p* < 0.001, adjusted R^2^ = 0.104]. When current psychiatric disorder was excluded from the model, mental health literacy remained unassociated with EPDS total scores (B = 0.017, 95% CI: −0.446 to 0.479, *p* = 0.944), and the findings for income, trimester and pregnancy planning were unchanged.

Residual diagnostics did not indicate departures from the assumptions of linear regression. In the complete-case analysis (*n* = 521), no problematic multicollinearity or highly influential observations were identified (maximum VIF = 2.15; maximum Cook’s distance = 0.093).

### 3.6. Exploratory Subscale Analyses

Exploratory subscale analyses were conducted post hoc. In the joint subscale model, higher knowledge-oriented literacy was associated with higher EPDS total scores (B = 0.706, 95% CI: 0.214 to 1.199, *p* = 0.005; Holm-adjusted *p* = 0.015), whereas higher resource-oriented literacy was associated with lower EPDS total scores (B = −0.564, 95% CI: −1.036 to −0.092, *p* = 0.019; Holm-adjusted *p* = 0.038); in the complete-case data, the addition of the subscales increased the explained variance by approximately 1.9%. Belief-oriented literacy was not associated with EPDS total scores (B = −0.281, 95% CI: −0.731 to 0.169, *p* = 0.220).

When each subscale was entered separately with the same covariates, the knowledge-oriented association remained nominally significant (B = 0.506, 95% CI: 0.034 to 0.977, *p* = 0.036), whereas the resource-oriented association was attenuated and no longer significant (B = −0.385, 95% CI: −0.839 to 0.068, *p* = 0.096); belief-oriented literacy remained non-significant (*p* = 0.264).

None of the subscales was independently associated with EPDS ≥ 10 screening positivity. In the ≥13 sensitivity analysis, a nominal negative association was observed for resource-oriented literacy (aOR = 0.809, 95% CI: 0.655 to 0.998, *p* = 0.048), which did not remain after Holm correction (Holm-adjusted *p* = 0.144). Complete-case fit statistics indicated acceptable calibration for both subscale models (EPDS ≥ 10: Hosmer–Lemeshow χ^2^(8) = 9.424, *p* = 0.308, Nagelkerke R^2^ = 0.142; EPDS ≥ 13: χ^2^(8) = 6.572, *p* = 0.583, Nagelkerke R^2^ = 0.136) (Table S2).

## 4. Discussion

The present study examined overall and domain-specific mental health literacy in relation to antenatal depressive symptom severity and EPDS-defined screening positivity. Neither the prespecified primary analysis nor the secondary screening analysis supported an association between the MHLS total score and the corresponding EPDS outcome after adjustment. Third-trimester and unplanned pregnancies were associated with higher EPDS scores and greater odds of EPDS ≥ 10 screening positivity, whereas income below expenses was associated only with higher continuous EPDS scores. Exploratory subscale analyses identified opposing and model-dependent associations for knowledge- and resource-oriented literacy; these findings should be regarded as hypothesis-generating rather than confirmatory.

### 4.1. Antenatal Depression in Pregnant Women

In the present study, 41.9% of the participants screened positive for elevated depressive symptoms at the EPDS ≥ 10 threshold. This finding represents the proportion of women with EPDS-defined screening positivity rather than the clinical prevalence of major depressive disorder, because the EPDS is a screening instrument and does not establish a psychiatric diagnosis [17,23]. Although systematic reviews and meta-analyses have also documented a substantial burden of depressive symptoms during pregnancy, reported estimates vary considerably across countries and study populations [10,11,12,13]. The relatively high proportion observed in the present study should therefore be interpreted in light of differences in sampling settings, sociodemographic characteristics, gestational stages and EPDS cut-off values rather than as evidence of a higher clinical prevalence.

The adjusted analyses revealed different patterns across the outcomes examined. Income below expenses was associated with higher continuous EPDS scores but was not significantly associated with EPDS ≥ 10 screening positivity. Third-trimester pregnancy was associated with higher continuous EPDS scores and greater odds of EPDS ≥ 10 screening positivity; however, the association did not reach statistical significance when the more stringent EPDS ≥ 13 threshold was used. In contrast, unplanned pregnancy was consistently associated with higher EPDS scores and greater odds of screening positivity at both EPDS thresholds. These results indicate statistical associations and should not be interpreted as evidence that income, gestational stage or pregnancy-planning status causes antenatal depressive symptoms.

The associations observed for financial disadvantage and unplanned pregnancy are broadly consistent with systematic reviews identifying low socioeconomic status, financial difficulties and unplanned pregnancy as important correlates of antenatal depression [11,18,30]. Multicenter and Türkiye-based studies have similarly reported associations between antenatal depressive symptoms and socioeconomic or pregnancy-related characteristics [31,32]. Financial strain may coincide with chronic stress and reduced access to practical or psychosocial resources, while an unplanned pregnancy may be accompanied by uncertainty and insufficient emotional or practical preparation. However, these possible pathways were not directly measured in the present study and should be regarded as explanatory hypotheses rather than demonstrated mechanisms.

The third-trimester finding may reflect changes in physical discomfort, concerns about childbirth and increasing practical and emotional demands as pregnancy progresses. Nevertheless, the absence of a statistically significant trimester association at the EPDS ≥ 13 threshold indicates that this finding was sensitive to outcome definition and should be interpreted cautiously. Overall, the results support the importance of considering pregnancy-planning status, gestational stage and socioeconomic circumstances when assessing antenatal depressive symptoms. Longitudinal studies that measure social support, stressful life events, relationship difficulties, financial strain and mental health service use are needed to clarify the temporal and explanatory pathways underlying these associations.

### 4.2. Mental Health Literacy Patterns Among Pregnant Women

In the present study, mental health literacy varied across some sociodemographic and pregnancy-related characteristics. Pregnant women with higher educational attainment had higher overall mental health literacy scores. This finding may reflect greater opportunities to access, understand and use health-related information. However, the observed group difference was based on an unadjusted comparison and should not be interpreted as evidence that education independently improves mental health literacy. Previous research indicates that perinatal mental health literacy encompasses multiple components, including symptom recognition, beliefs and attitudes concerning mental disorders, knowledge of available support and willingness to seek professional help [4,19]. Studies conducted among pregnant and postpartum women have also reported limitations in the recognition of perinatal mental health problems and knowledge of appropriate sources of support, with literacy levels varying according to sociodemographic characteristics [5,20,21].

Beyond general educational attainment, psychoeducational interventions aimed at improving mental health literacy among pregnant women have been emphasized as an important strategy [16,19,21]. Nevertheless, the effects of such interventions may differ across mental health literacy dimensions. A recent cluster-randomized trial found that antenatal psychoeducation improved overall postpartum depression literacy, symptom recognition and access to information but did not significantly improve attitudes towards professional help-seeking [16]. Thus, improving knowledge or awareness of resources does not necessarily result in changes in help-seeking attitudes or behavior.

The belief-oriented dimension of mental health literacy assesses traditional beliefs about mental illness and is reverse scored [2]. The score pattern observed among pregnant women reporting current medication use may be compatible with fewer traditional beliefs about mental illness and greater acceptance of contemporary treatment approaches. Previous contact with healthcare professionals could have contributed to this finding, as women using medication also exhibited higher levels of knowledge-oriented mental health literacy. However, the study did not establish whether current medication use represented psychotropic treatment, and the frequency or nature of participants’ previous contact with healthcare professionals was not assessed. This interpretation should therefore be regarded as a possible explanation rather than a demonstrated mechanism.

Furthermore, first-time pregnant women demonstrated higher levels of resource-oriented mental health literacy. This may reflect their greater tendency to seek information from healthcare providers or to rely on routine antenatal care and the experiences of others during pregnancy. However, participants’ frequency of information seeking and their reasons for selecting particular information sources were not measured. Previous studies have shown that pregnant women may hold misconceptions or traditional beliefs about mental illness and may primarily seek support from informal sources, including close social networks and spiritual or religious advisors [19,21]. Women experiencing psychological difficulties during pregnancy may nevertheless need support from healthcare professionals, although stigma, limited awareness of services and healthcare-system barriers can restrict the use of professional mental healthcare [6,7,15]. The present findings may therefore indicate that contact with different information and support sources is associated with differences in women’s mental health knowledge and beliefs. However, the cross-sectional design does not establish that changes in information sources lead to changes in mental health literacy.

The differences according to education, current medication use and pregnancy number were derived from unadjusted subgroup comparisons. They may therefore reflect confounding or multiple comparisons and should be interpreted as descriptive and exploratory rather than as independent associations. Antenatal educational initiatives may reasonably aim to improve symptom recognition, awareness of reliable information resources and knowledge of referral pathways; however, the present study did not evaluate an educational intervention and cannot establish that increasing mental health literacy reduces antenatal depressive symptoms.

### 4.3. Associations Between Mental Health Literacy and Antenatal Depressive Symptoms

In the present study, no significant association was found between mental health literacy and antenatal depression risk, and mental health literacy was not an independent predictor of antenatal depression risk. This finding suggests that mental health literacy alone is not an independent determinant of antenatal depression risk. It may be interpreted in light of the multifactorial nature of antenatal depression, which is influenced by the complex interplay of biological, psychosocial, and socioeconomic factors [6,10,11,14]. Accordingly, rather than exerting a direct effect on the risk of antenatal depression, mental health literacy may influence the risk through interactions with other risk factors or via indirect mechanisms. However, these potential pathways were not examined in the present study. Future research employing mediation analyses or structural equation modeling is warranted to investigate these mechanisms.

Kukoyi et al. (2023) found that pregnant women with poor depression literacy had a greater tendency to experience depressive symptoms [33]. The discrepancy between these findings and those of the present study may be explained by differences in the constructs examined, as the previous study focused specifically on depression literacy and depressive symptoms, whereas the present study evaluated overall mental health literacy in relation to both depressive symptom severity and EPDS-defined screening positivity. Depression-specific literacy may be more closely related to the recognition and interpretation of depressive symptoms than a broader measure encompassing knowledge, beliefs and awareness of mental health resources.

The instrument used in this study assessed general mental health literacy rather than literacy specifically tailored to the antenatal period. However, the antenatal period is characterised by distinctive psychosocial challenges, including adaptation to pregnancy and motherhood, concerns about childbirth and fetal well-being and changes in close relationships [6,12,34]. Evidence from clinical trials indicates that antenatal education and midwifery-led care may improve maternal anxiety and depressive symptoms, although these interventions are not equivalent to assessing or improving mental health literacy [7,35]. For example, Mirsalimi et al. (2020) developed the Postpartum Depression Literacy Scale to assess literacy specifically related to postpartum depression [36]. Therefore, the development or validation of measurement instruments specifically designed to assess antenatal mental health literacy may facilitate a more contextually appropriate evaluation of its association with antenatal depressive symptoms.

In addition to mental health literacy itself, translating knowledge into appropriate help-seeking behaviour may be an important mechanism linking mental health literacy with antenatal mental health outcomes. Having knowledge about antenatal mental health does not necessarily translate into professional help-seeking behaviour. For example, antenatal psychoeducation has been shown to improve symptom recognition and access to information without producing a corresponding significant improvement in attitudes towards professional help-seeking [16,37]. Pregnant women may not adequately utilise professional mental health services because of concerns about judgement and stigma, pregnancy-related challenges, limited awareness of available services, financial pressure, insufficient partner support and barriers within the healthcare system [6,7,19,21,34,37,38].

These barriers may offer one possible explanation for why higher knowledge-oriented mental health literacy was associated with higher levels of depressive symptoms in the exploratory joint subscale model. Knowledge about mental health may increase awareness and recognition of antenatal depressive symptoms; however, without translating this knowledge into appropriate help-seeking behaviour, greater awareness may not be accompanied by lower symptom levels. An alternative explanation is reverse directionality: women experiencing more depressive symptoms may seek additional mental health information and consequently acquire greater knowledge. Because help-seeking behaviour and the timing of information acquisition were not assessed, and because the data were collected cross-sectionally, these potential explanations could not be examined directly.

The existing literature also suggests that women’s social environments influence how they recognize mental health difficulties and whether they seek professional support. Pregnant women may rely on close social networks and other informal sources while making limited use of professional perinatal mental health services [6,19,38]. Increasing mental health literacy at the community level has therefore been emphasized as a potentially important strategy for promoting perinatal mental health. Taken together, these findings suggest that individual-level mental health literacy and high levels of mental health knowledge alone may not be sufficient to ensure timely access to appropriate maternal mental health services.

In the joint exploratory subscale model, higher resource-oriented mental health literacy was associated with lower continuous EPDS scores. However, this association was attenuated and did not remain statistically significant when resource-oriented literacy was examined separately with the same covariates. Resource-oriented literacy was also not associated with EPDS ≥ 10 screening positivity. Although a nominal association was observed at the EPDS ≥ 13 threshold, it did not remain statistically significant after correction for multiple comparisons. The resource-oriented finding should therefore be regarded as model-dependent and exploratory rather than as evidence that access to mental health resources protects women against antenatal depression.

One limitation of the present study is that it did not comprehensively assess participants’ access to information sources, the quality of these sources or their ability to apply the information obtained. Resource-oriented mental health literacy involves awareness of and access to appropriate mental health information and professional support resources [2,4]. Higher levels of mental health knowledge do not necessarily ensure appropriate help-seeking, just as lower levels of knowledge do not inevitably lead to inappropriate help-seeking. Rather, the availability and quality of information sources and individuals’ ability to apply their knowledge appropriately may be relevant to whether professional support is obtained [19,22,38]. Given the importance of informal, healthcare and digital information sources during pregnancy, future research should investigate how digital and community mental health literacy are associated with information appraisal, service awareness and professional help-seeking. Interventions intended to enhance digital or community mental health literacy may warrant evaluation, but their effects on antenatal depressive symptoms cannot be inferred from the present study.

Although the knowledge-, resource- and belief-oriented dimensions represent components of the same construct, their associations were not uniform. In the joint exploratory linear model, knowledge-oriented literacy was associated with higher continuous EPDS scores, resource-oriented literacy was associated with lower scores and belief-oriented literacy was not associated with EPDS scores. However, the resource-oriented association was attenuated when the subscales were examined separately, and none of the three dimensions was associated with EPDS ≥ 10 screening positivity. These post hoc findings indicate that the dimensions of mental health literacy may differ in their associations with antenatal depressive symptom severity, but they do not demonstrate corresponding differences in the likelihood of screening positivity. They should therefore be considered hypothesis-generating and require replication in prospectively designed studies.

The present study highlights the need to reconsider the conditions under which different dimensions of mental health literacy are associated with antenatal mental health outcomes. Future longitudinal studies should examine the temporal and potentially bidirectional relationships among mental health literacy, depressive symptoms, information-seeking and professional help-seeking. Mediation and interaction models may then help clarify whether social support, service accessibility and the quality of mental health information explain or modify these associations.

### 4.4. Limitations and Strengths

The findings of this study should be interpreted in light of several limitations. First, the cross-sectional design does not permit conclusions about temporal direction or causality. Therefore, the findings cannot establish whether mental health literacy precedes changes in depressive symptoms or whether women experiencing more symptoms subsequently acquire greater mental health knowledge or seek additional information. Second, although consecutive recruitment reduced discretionary participant selection within the study setting, the sample was drawn from a single university hospital. The findings may therefore not be fully generalizable to pregnant women receiving care in different geographical regions, primary care settings or healthcare systems.

The EPDS is a screening instrument rather than a diagnostic assessment; consequently, EPDS-defined screening positivity should not be interpreted as a DSM-5 diagnosis of major depressive disorder with peripartum onset. In addition, the use of self-reported measures may have introduced response or recall bias. Potentially relevant factors, including social support, help-seeking behavior, access to mental health services and the quality and use of mental health information sources, were not comprehensively assessed. Their possible contribution to the observed associations and potential indirect pathways could therefore not be evaluated. Finally, the subscale analyses were exploratory, and some associations varied across model specifications. These findings should therefore be regarded as hypothesis-generating and require replication.

The study also has several strengths. It included a relatively large sample of women in the second and third trimesters and assessed mental health literacy using both an overall score and its knowledge-, belief- and resource-oriented dimensions. The analyses considered depressive symptoms as both a continuous outcome and EPDS-defined screening positivity, thereby avoiding reliance on a single outcome definition. Multivariable models adjusted for relevant sociodemographic, obstetric and health-related characteristics, while multiple imputation was used to address missing data. Sensitivity analyses using an alternative EPDS threshold, separate subscale models and correction for multiple comparisons further supported a transparent and cautious evaluation of the robustness of the findings. Overall, the study found no evidence of an independent association between total mental health literacy and antenatal depressive symptoms in this sample while identifying domain-specific patterns that may inform future research.

Future longitudinal and multicenter studies should use repeated assessments across pregnancy to clarify the temporal and potentially bidirectional relationships between mental health literacy dimensions, information-seeking, help-seeking and depressive symptoms. Research should also examine how these dimensions interact with pregnancy-planning status, gestational stage, socioeconomic circumstances, social support and access to mental health services. The development or pregnancy-specific adaptation and validation of mental health literacy instruments may improve the assessment of literacy within the antenatal context, as existing measures generally assess broader mental health literacy or literacy related to the postpartum period [19,36]. Domain-specific hypotheses should be specified prospectively, and intervention studies should be considered after the exploratory subscale associations identified in this study have been replicated.

## 5. Conclusions

A substantial proportion of pregnant women in this study (41.9%) screened positive for elevated antenatal depressive symptoms, reinforcing the importance of routine mental health screening throughout pregnancy. Overall mental health literacy was not independently associated with depressive symptom severity or EPDS ≥ 10 screening positivity; therefore, the prespecified primary hypothesis was not supported, and the secondary screening analysis was also null. Third-trimester pregnancy and unplanned pregnancy were associated with higher odds of screening positivity, while income lower than expenses was associated with greater symptom severity. Exploratory analyses showed opposing and model-dependent associations across mental health literacy dimensions: knowledge-oriented literacy was associated with higher EPDS scores and resource-oriented literacy with lower scores in the joint model, but the resource-oriented association was attenuated when examined separately, and no subscale was associated with EPDS ≥ 10 screening positivity. These findings do not establish causal or protective effects of mental health literacy. Clinically, routine antenatal screening should be accompanied by particular attention to unplanned pregnancy, later gestation and socioeconomic disadvantage, together with accessible referral pathways for women screening positive.

## Figures and Tables

**Table 1:** Distribution of sociodemographic and pregnancy characteristics (*n* = 568).

**Sociodemographic Characteristics**	**n**	**%**
Education (n = 564)		
Literate—Primary degree	230	40.8
High school degree	210	37.2
Bachelor’s and post-graduate degree	124	22.0
Income (*n* = 558)		
Exceeds expenses	97	17.4
Equal to expenses	273	48.9
Lower than expenses	188	33.7
**Pregnancy Characteristics**	**n**	**%**
Period of trimester(*n* = 558)		
Second (20–27 weeks)	119	21.3
Third (Above 27 weeks)	439	78.7
Pregnancy planning status (*n* = 562)	384	68.3
Number of pregnancies (*n* = 552)		
1st	187	33.9
2nd	181	32.8
3rd or later	184	33.3
Previous miscarriage or pregnancy loss (*n* = 562)		
At least one	181	32.2
None	381	67.8
Infertility treatment (*n* = 564)		
Yes	38	6.7
Intrauterine insemination	31	5.5
In vitro fertilization	7	1.2
Current physical disorder	47	8.3
Current psychiatric disorder	6	1.1
Current medication except for supplements (*n* = 563)		
Yes	114	20.2
No	449	79.8
Receiving education about pregnancy		
Yes (from midwives, nurses, physicians, local government health departments, paid services from non-government medical staff)	30	5.3
Receiving information about pregnancy ^1^		
From internet	457	80.5
From medical staff	282	49.6
From acquaintances	64	11.3
From visual media—television	37	6.5
From written media—newspaper/journal	21	3.7

Note. Percentages were calculated using the valid number of responses for each variable. ^1^ Multiple responses were permitted; percentages were calculated using the total sample (*n* = 568).

**Table 2:** Unadjusted comparisons of EPDS scores by sociodemographic and pregnancy-related characteristics (*n* = 568).

**Scale Score Averages**	$\bar{X}$	**SD**	**Min**	**Max**	**Range**		
Edinburgh Postnatal	8.92	5.68	0.00	30.00	0–30		
Depression Scale							
**Variables**	***n***	$\bar{X}$	**Ss**	**KW**	**MWU**	**Z**	***p* value**
Period of pregnancy							
2nd trimester	119	7.46	5.36		20,911.0	−3.345	0.001
3rd trimester	439	9.30	5.69				
Income							
Exceeds expenses	97	8.96	5.73	32.082			0.001
Equal to expenses	273	7.64	5.05				
Lower than expenses	188	10.71	5.88				
Planned pregnancy							
Yes	384	8.15	5.27		25,976.0	−4.586	0.001
No	178	10.57	6.04				

Abbreviation: EPDS: Edinburgh Postnatal Depression Scale. SD: standard deviation; KW: Kruskal–Wallis Test. MWU: Mann–Whitney U Test. Note. Due to space limitations, only the independent demographic factors showing statistically significant differences are presented in the table.

**Table 3:** Mental health literacy scores and unadjusted comparisons by sociodemographic and pregnancy-related characteristics (*n* = 568).

**Scale Score Averages**			$\bar{X}$	**SD**	**Min**	**Max**	**Range**	
Mental Health Literacy Scale			12.90	4.30	0.00	22.00	0–22	
Knowledge-oriented			6.71	2.62	0.00	10.00	0–10	
Belief-oriented			3.38	2.45	0.00	8.00	0–8	
Resource-oriented			2.82	1.36	0.00	4.00	0–4	
**Variables**		***n***	$\bar{X}$	**Ss**	**KW**	**MWU**	**Z**	***p* value**
MHLS Total	Education							
	Literate—Primary school	230	11.66	4.46	30.470			0.001
	High school	210	13.52	4.05				
	Bachelor’s degree and higher	124	14.17	3.86				
Knowledge-oriented	Education							
	Literate—Primary school	230	5.87	2.72	64.115			0.001
	High school	210	6.81	2.36				
	Bachelor’s degree and higher	124	8.09	2.22				
	Current medication except for supplements							
	Yes	114	7.14	2.51		22,307.5	−2.136	0.033
	No	449	6.59	2.64				
Belief-oriented	Current medication except for supplements							
	Yes	114	2.85	2.21		21,777.0	−2.480	0.013
	No	449	3.52	2.49				
Resource- oriented	Education							
	Literate—Primary school	230	2.56	1.49	11.620			0.003
	High school	210	3.04	1.20				
	Bachelor’s degree and higher	124	2.93	1.27				
	Number of pregnancies							
	1st	187	3.02	1.28	7.834			0.020
	2nd	181	2.64	1.38				
	3rd and more	184	2.77	1.40				

Abbreviations. MHLS: Mental Health Literacy Scale; SD: standard deviation; KW: Kruskal–Wallis test; MWU: Mann–Whitney U test. Note. Only variables showing statistically significant differences are presented. Medication use excludes vitamin and mineral supplements. Valid sample sizes are shown for each variable.

**Table 4:** Spearman correlations between mental health literacy and EPDS scores (*n* = 568).

Scale Scores		MHLS	Knowledge-Oriented	Belief-Oriented	Resource-Oriented
**EPDS total**	rho	−0.014	0.040	−0.023	−0.063
	*p*	0.438	0.345	0.585	0.132

Abbreviation: EPDS: Edinburgh Postnatal Depression Scale, MHLS: Mental Health Literacy Scale. rho: Spearman’s correlation.

**Table 5:** Multivariable linear regression of factors associated with EPDS total scores (*n* = 568).

Variable	B	SE	95% CI	*p*
Mental health literacy, per 1SD increase	0.011	0.236	−0.451 to 0.473	0.963
Age, years	−0.084	0.048	−0.178 to 0.010	0.081
Second pregnancy	0.530	0.596	−0.639 to 1.699	0.374
Third or later pregnancy	0.050	0.716	−1.354 to 1.454	0.945
Previous miscarriage or pregnancy loss	0.904	0.530	−0.136 to 1.943	0.088
Literate–primary school	0.128	0.665	−1.176 to 1.432	0.848
High school	0.172	0.644	−1.090 to 1.434	0.789
Income equal to expenses	−1.280	0.650	−2.555 to −0.006	0.049
Income lower than expenses	1.548	0.687	0.202 to 2.894	0.024
Third trimester	1.572	0.572	0.452 to 2.693	0.006
Unplanned pregnancy	2.127	0.524	1.100 to 3.154	<0.001
Current psychiatric disorder	2.881	2.293	−1.616 to 7.377	0.209
Current physical disorder	−0.131	0.841	−1.780 to 1.518	0.877

Abbreviations. EPDS: Edinburgh Postnatal Depression Scale; SE: standard error; CI: confidence interval. Note. Reference categories: first pregnancy; bachelor’s degree or higher; income exceeds expenses; second trimester; planned pregnancy; no previous miscarriage or pregnancy loss; no current psychiatric disorder; no current physical disorder. All covariates were entered simultaneously. Estimates were pooled across 20 imputed datasets using Rubin’s rules.

**Table 6:** Multivariable logistic regression of factors associated with EPDS ≥ 10 screening positivity (*n* = 568).

Variable	aOR	95% CI	*p*
Mental health literacy, per 1 SD increase	1.093	0.911 to 1.312	0.337
Age, years	0.978	0.942 to 1.015	0.231
Second pregnancy	1.262	0.793 to 2.006	0.326
Third or later pregnancy	0.879	0.504 to 1.534	0.651
Previous miscarriage or pregnancy loss	1.399	0.930 to 2.105	0.107
Literate–primary school	1.274	0.757 to 2.143	0.362
High school	1.282	0.776 to 2.119	0.333
Income equal to expenses	0.615	0.375 to 1.008	0.054
Income lower than expenses	1.525	0.914 to 2.546	0.106
Third trimester	1.774	1.123 to 2.805	0.014
Unplanned pregnancy	1.975	1.330 to 2.935	0.001
Current psychiatric disorder	3.368	0.525 to 21.597	0.200
Current physical disorder	0.909	0.470 to 1.758	0.777

Abbreviations. aOR: adjusted odds ratio; CI: confidence interval; EPDS: Edinburgh Postnatal Depression Scale. Note. Reference categories as in Table 5. EPDS ≥ 10 indicates screening positivity for elevated depressive symptom risk, not a clinical diagnosis. All covariates were entered simultaneously. Estimates were pooled across 20 imputed datasets using Rubin’s rules.

## Data Availability

All de-identified data files underlying the findings of this study are available from the corresponding author upon reasonable request. The data are not publicly available because participants were not informed that their data would be deposited in a public repository, and specific consent for unrestricted public sharing was not obtained.

## Supplementary Materials

The following supporting information can be downloaded at: https://www.mdpi.com/article/10.3390/healthcare14162545/s1, Table S1: Multivariable models; Table S2: Exploratory associations between mental health literacy subscales and antenatal depressive symptoms (*n* = 568).
