# Supplementary material for: Antenatal Depression Risk and Mental Health Literacy: A Correlational Study Among Second- and Third-Trimester Pregnancies
Source: Healthcare (Basel). 2026 Aug 14;14(16):2545. doi: 10.3390/healthcare14162545 (PMC13512527; doi:10.3390/healthcare14162545)
Supplement: Supplementary file 1 [file healthcare-14-02545-s001.zip › healthcare-4424607-supplementary.pdf]

Table S1. Multivariable models.

Model	Dependent variable	Main independent variable	n
Model 1 — Primary	EPDS total score	MHLS total score	568
Model 2 — Secondary	EPDS ≥10 screening positivity	MHLS total score	568 (238)
Model 3 — Sensitivity	EPDS ≥13 screening positivity	MHLS total score	568 (141)
Model 4 — Sensitivity	EPDS total score	MHLS total score	521
Model 5 — Sensitivity	EPDS total score	MHLS total score	568
Model 6 — Exploratory	EPDS total score	MHLS three subscales	568
Model 7 — Exploratory	EPDS ≥10 screening positivity	MHLS three subscales	568 (238)
Model 8 — Exploratory	EPDS ≥13 screening positivity	MHLS three subscales	568 (141)
Model 9 — Sensitivity	EPDS total score	Knowledge-oriented subscale	568
Model 10 — Sensitivity	EPDS total score	Belief-oriented subscale	568
Model 11 — Sensitivity	EPDS total score	Resource-oriented subscale	568

Abbreviation. MHLS: Mental Health Literacy Scale. *Note.* Values in parentheses indicate the number of participants screening positive. Models 1, 4–6 and 9–11 are linear regressions; Models 2, 3, 7 and 8 are binary logistic regressions. All models were adjusted for age, number of pregnancies, history of miscarriage or pregnancy loss, education level, income level, trimester, pregnancy planning status, current mental and physical disorders. Model 4 is a complete-case analysis; missing data were handled by multiple imputation in all other models. Model 5 was repeated with current mental disorder excluded.

Table S2. Exploratory associations between mental health literacy subscales and antenatal depressive symptoms (n = 568).

Subscale, per 1 SD increase	EPDS total B (95% CI)	p	Holm p	EPDS ≥10 aOR (95% CI)	p	Holm p	EPDS ≥13 aOR (95% CI)	p	Holm p
**Knowledge-oriented**	0.706 (0.214 to 1.199)	0.005	0.015	1.216 (0.996 to 1.486)	0.055	0.165	1.146 (0.910 to 1.443)	0.246	0.492
**Belief-oriented**	−0.281 (−0.731 to 0.169)	0.220	0.220	0.988 (0.825 to 1.182)	0.892	0.892	0.893 (0.727 to 1.096)	0.280	0.492
**Resource-oriented**	−0.564 (−1.036 to −0.092)	0.019	0.038	0.926 (0.767 to 1.119)	0.427	0.854	0.809 (0.655 to 0.998)	0.048	0.144

Abbreviations. aOR: adjusted odds ratio; CI: confidence interval; EPDS: Edinburgh Postnatal Depression Scale; SD: standard deviation. *Note.* All three subscales were entered simultaneously without the total scale score. All models were adjusted for the covariates listed in Table 5. Holm p denotes p values adjusted for multiple comparisons across the three subscales using the Holm method. Estimates were pooled across 20 imputed datasets using Rubin’s rules. These analyses were exploratory.
